# Risk Factors for Postoperative Intra-Abdominal Abscess in Pediatric Perforated Appendicitis Following Laparoscopic Appendectomy: A Multicenter Analysis

**DOI:** 10.3390/children11111385

**Published:** 2024-11-14

**Authors:** Joonhyuk Son, Ji-Won Han, Chaeyoun Oh

**Affiliations:** 1 Department of Pediatric Surgery, Hanyang University College of Medicine, Seoul 04763, Republic of Korea; cross0911@hanyang.ac.kr; 2 Department of Surgery, Ewha Womans University Seoul Hospital, Seoul 07804, Republic of Korea; 01120s@eumc.ac.kr; 3 Division of Pediatric Surgery, Department of Surgery, Korea University College of Medicine, Korea University Ansan Hospital, Ansan 15355, Republic of Korea

**Keywords:** abscess, appendicitis, children, infections, predictors

## Abstract

Background: Perforated appendicitis in children is a frequently encountered and significant surgical condition. The treatment of choice is laparoscopic appendectomy, but this carries a risk of postoperative intra-abdominal abscess (IAA). The purpose of this study was to determine risk factors linked to the occurrence of IAA following laparoscopic surgery in pediatric perforated appendicitis. Methods: This retrospective cohort study analyzed 137 children with perforated appendicitis who received laparoscopic appendectomy at four tertiary hospitals between March 2018 and December 2022. Data on patient demographics, preoperative clinical characteristics, and surgical details were collected. Independent risk factors for IAA formation were determined using logistic regression analysis. Results: The overall incidence of postoperative IAA was 10.9%. Prolonged symptom duration and elevated CRP levels were associated with higher IAA rates. Patients who developed IAAs experienced prolonged postoperative fevers and longer hospital stays. Significant risk factors for IAA identified through multivariable analysis included a higher severity grade of appendicitis (≥Grade IV, OR 5.9, *p* = 0.034) and the presence of a free appendicolith during surgery (OR 5.549, *p* = 0.01). Of the patients who developed IAAs, nine (60%) improved with conservative treatment, while six (40%) required invasive procedures. Conclusions: A higher severity grade of appendicitis (≥Grade IV) and the presence of a free appendicolith are significant predictors of postoperative IAAs in pediatric perforated appendicitis. Recognizing these factors can help guide clinical management and postoperative care, potentially reducing the incidence of this complication.

## 1. Introduction

Acute appendicitis (AA) is among the most common surgical emergencies in children, necessitating prompt intervention to avert complications. Recently, for uncomplicated appendicitis, non-operative treatments such as antibiotic therapy have gained attention as potential alternatives, offering less-invasive options with promising results in selected cases [1]. Perforated appendicitis in children is known to account for about 20% of all AA cases [2,3,4]. In the treatment of perforated appendicitis in children, the most widely accepted treatment is a combination of antibiotics and appendectomy. However, there is ongoing debate regarding early appendectomy versus delayed appendectomy [5].

Laparoscopic appendectomy is the preferred treatment due to its minimally invasive nature and associated benefits, such as reduced postoperative pain and quicker recovery times compared to the open approach [6,7]. Despite these advantages, some researchers argue that laparoscopic appendectomy for children with perforated appendicitis presents a higher risk of postoperative complications, particularly the development of intra-abdominal abscesses (IAAs), when compared to the open approach [8,9,10]. If an IAA occurs, the patient may experience prolonged hospitalization and may even require readmission. Additionally, large abscesses may require percutaneous or surgical drainage, which is a particularly burdensome complication [11]. The reported incidence of IAAs after appendectomy for perforated appendicitis in children varies greatly across the literature but is reported to be relatively common, with incidence rates ranging from about 14% to 18% [12,13,14].

This study aimed to identify risk factors linked to the occurrence of postoperative IAAs in children undergoing laparoscopic appendectomy for perforated appendicitis.

## 2. Materials and Methods

**Figure 1:**
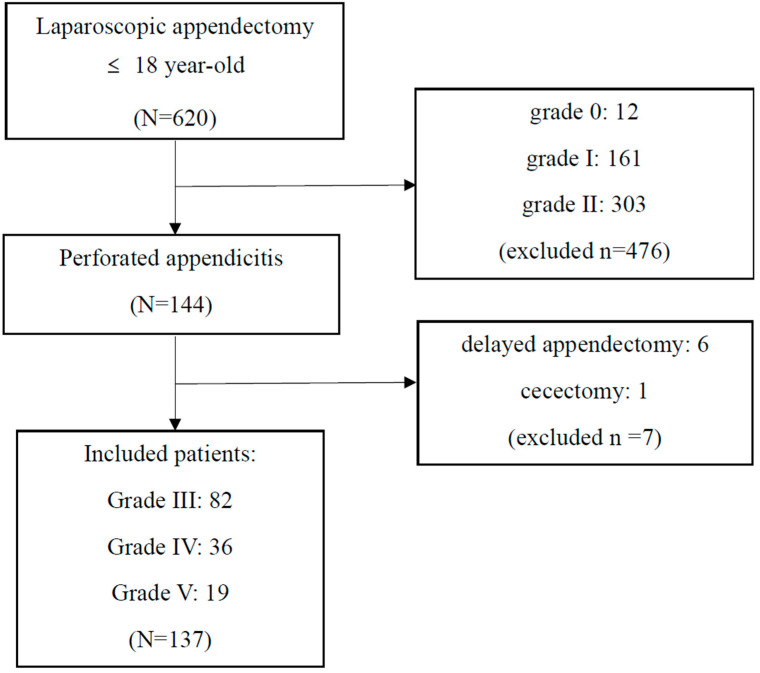
Flowchart of the included patients.

This retrospective cohort study analyzed patients diagnosed with perforated appendicitis at four tertiary hospitals in Korea from March 2018 to December 2022. Surgeries at hospitals A and B were conducted by the corresponding author, while those at hospitals C and D were performed by a co-author serving as the attending surgeon at each hospital. Patients included in this study were under 19 years of age at the time of surgery, all of which were operated on laparoscopically, without any conversions to open surgery. The study included patients confirmed to have perforated AA who underwent early appendectomy. During the study period, a total of 620 patients received laparoscopic appendectomy. After excluding patients with negative appendectomy, 608 patients remained, of whom 144 (23.7%) had perforated appendicitis. Of these, 137 patients who did not undergo delayed appendectomy or cecectomy were incorporated into the final analysis (Figure 1).

The study analyzed variables such as gender, age, body mass index, duration of symptoms, white blood cell (WBC) count, neutrophil count, C-reactive protein (CRP) level, diagnostic tools, methods of laparoscopic approach, severity grade of AA, appendicolith presence on imaging, presence of free appendicolith at the time of surgery, operation time, whether a peritoneal drain (PD) was placed during surgery, postoperative course, and complications, including IAAs. The severity grade of AA was classified according to the American Association for the Surgery of Trauma Emergency General Surgery (AAST-EGS) Grading Scale [15], with Grades 3, 4, and 5 classified as perforated appendicitis. The attending surgeon determined the grading of each patient.

The treatment strategy across the four hospitals was to administer broad-spectrum intravenous (IV) antibiotics immediately after diagnosis if perforated appendicitis was suspected on preoperative imaging. If no perforation was suspected, prophylactic antibiotics were administered before surgery. Postoperatively, broad-spectrum IV antibiotics were continued until abdominal pain and fever subsided, and patients were discharged once symptoms improved. Patients without complications were followed up approximately one week post-discharge in an outpatient setting.

In this study, an IAA was defined as a cystic collection of dirty debris with an enhanced rim or symptomatic fluid collections (≥1 cm with a fever of 38 °C lasting more than 3 days) occurring at least 5 days postoperatively. IAAs were diagnosed using abdominal ultrasound or computed tomography (CT). A single lesion larger than 20 cm^2^ was classified as a large abscess, and percutaneous drainage (PCD) was performed when feasible [16].

We analyzed categorical variables using the chi-square test or Fisher’s exact test, and evaluated continuous variables with the Mann–Whitney U test. Comparisons were made between patients with and without IAAs. Logistic regression analysis was conducted to identify independent risk factors for IAA. To perform the multivariable logistic regression analysis, we selected variables that were statistically significant in the univariable analysis, excluding those influenced by IAA treatment factors (e.g., hospital days after appendectomy, readmission). Continuous variables were analyzed using the binarization method (e.g., CRP level ≥ 10 mg/dL), and severity grading was transformed into categories such as Grade IV, Grade V, and Grade ≥ IV. A *p*-value less than 0.05 was considered statistically significant, and analyses were performed using SPSS software version 20 (SPSS Inc., Chicago, IL, USA).

This study was approved by the Institutional Review Board at Korea University Ansan Hospital (IRB File No. 2024AS0244).

## 3. Results

Among the 137 patients included in the study, 76 (55.5%) were male, with a median age of 11.19 years at the time of surgery. In terms of severity, 82 patients (59.9%) had Grade III appendicitis, 36 patients (26.3%) had Grade IV, and 19 patients (13.8%) had Grade V. A total of 56 patients (40.9%) underwent single-port laparoscopic surgery, and 85 patients (62%) had a PD placed during surgery. The median postoperative hospital stay was 3 days, and 114 patients (83.2%) were prescribed oral antibiotics upon discharge. Postoperative complications occurred in 28 patients (20.4%), with IAAs occurring in 15 patients (10.9%) (Table 1). Although no patients had significant underlying diseases, four patients had unique conditions: nutcracker syndrome, congenital bilateral absence of the arms, tic disorder, and muscular dystrophy.

The overall incidence of IAA was 10.9%, and the rates of IAA were similar across hospitals. No significant differences in gender, age, or body weight were observed between the IAA and non-IAA groups. However, patients in the IAA group had a significantly longer median duration of symptoms to operation compared to those in the non-IAA group (58 vs. 38 h, *p* = 0.012). Preoperative fever and WBC counts were slightly higher in the IAA group, but these differences were not statistically significant. However, the IAA group had a significantly elevated median CRP level (10.6 vs. 4.33 mg/dL, *p* = 0.019). The presence of appendicolith on preoperative imaging (93.3% vs. 63.1%, *p* = 0.02) and free appendicolith observed during surgery (60% vs. 10.6%, *p* < 0.001) were significantly more common in the IAA group. The severity grade of appendicitis was also significantly higher in the IAA group, with a greater proportion of patients having Grade IV or V appendicitis. While there were no significant differences between the groups in terms of surgical approach, PD placement, or the administration of oral antibiotics at discharge, the IAA group exhibited a significantly higher rate of prolonged postoperative fever and experienced longer hospital stays (Table 2).

Multivariable logistic regression analysis identified a grade of AA ≥ IV (*p* = 0.034, Odds Ratio (OR) 5.9) and free appendicolith at surgery (*p* = 0.01, OR 5.549) as significant factors contributing to the development of IAAs (Table 3).

Details of the 15 patients who developed IAAs are summarized in Table 4. The median postoperative day of IAA diagnosis was 11 days (range: 5–62). Six patients (40%) were diagnosed with IAAs during their primary hospital admission, while nine patients (60%) were readmitted after being discharged and subsequently diagnosed with IAAs. These readmitted patients had a median hospital stay of 7 days (range: 3–17). Four patients with abscesses larger than 20 cm^2^ underwent PCD, while nine patients with smaller abscesses were successfully treated with IV broad-spectrum antibiotics. Two patients required reoperation due to retained appendicoliths, which were discovered on postoperative days 62 and 5, respectively. There were no recurrences of IAA in any patients.

## 4. Discussion

AA is a commonly occurring surgical condition in children, with a variable prevalence worldwide. In South Korea, the incidence of appendectomy in children under 18 was recently reported as 2.25 per 1000 people, with perforated cases ranging from 16.8% to 22.9% [2,3,4,17,18]. Variation in the rate of perforated appendicitis is influenced by geographic and socioeconomic factors, as well as differences in diagnostic criteria. This is further supported by reports from various children’s hospitals in the United States, indicating that perforation rates of AA among children have ranged from 20% to 76% [19]. We applied the AAST-EGS grading system to classify AA severity, which allows for more accurate outcome prediction and resource allocation based on clinical, imaging, and operative findings [15,20].

The mechanism behind postoperative IAA formation remains unclear. In children with uncomplicated appendicitis, the incidence of postoperative IAA is low, at approximately 1% [13]. However, it is relatively common in perforated appendicitis. Some earlier studies reported that, in cases of perforated appendicitis, laparoscopic appendectomy was associated with a higher rate of postoperative IAA compared to open appendectomy [21,22]. However, more recent studies have shown similar or even better outcomes with laparoscopy [23,24]. Due to the many advantages of laparoscopic surgery, most centers now prefer it for treating perforated appendicitis in children.

CT imaging is more effective than ultrasound in detecting appendicolith and diagnosing acute appendicitis [25,26]. Based on several studies, among children with acute appendicitis, ultrasound has reported sensitivity and specificity rates of 54–58% and 70–78% for detecting appendicolith, respectively [27,28], while CT shows a sensitivity of 65% and a specificity of 86% [29]. In the imaging studies of our research, the detection rates for appendicolith were 6/11 (54.5%) with ultrasound and 85/126 (67.5%) with CT.

In this study, the management of IAAs was determined by the size of the abscess; treatment options included PCD or conservative therapy. Two patients in our study underwent reoperation due to retained appendicoliths. Although it is uncommon for an appendicolith to be left behind after laparoscopic appendectomy, this complication can occur if the appendicolith is not identified and removed during surgery [30]. Retained appendicolith may lead to infection, with inflammation and infection potentially developing days or even years later. It is unclear whether all retained appendicoliths cause symptoms or whether appendicolith size is a factor. However, symptomatic retained appendicolith should be removed to prevent chronic infection [30,31]. In our study, two patients had retained appendicoliths identified on postoperative days 62 and 5, respectively, and both required surgical removal due to the inability to access the appendicolith nonoperatively. In the first case, a female patient aged 16 presented with severe Grade V generalized peritonitis during the initial surgery, making it difficult to clearly identify the appendix. As a result, an exposed appendicolith was left behind unknowingly. In the second case, a 6-year-old girl exhibiting Grade IV severity had an appendicolith located at the distal end of the appendix. During the initial surgery, the distal end of the appendix tore during traction, leaving a portion behind unknowingly, and the surgery was completed without recognizing this issue. Both patients underwent laparoscopic surgery, resulting in successful outcomes.

Is there a way to prevent or reduce the incidence of IAA? A 2019 systematic review reported no reduction in IAAs when peritoneal irrigation was performed during appendectomy [32]. Many surgeons insert a PD postoperatively to facilitate drainage of complicated fluid in cases of severe peritonitis. However, this practice has been reported to either have no effect or even increase the rate of IAA formation [33,34]. In our study, PDs were applied in 62% of patients. Although PD placement was more common in the IAA group, statistical analysis revealed no significant difference. Further investigation is needed to determine whether increased PD placement in the IAA group was due to appendicitis severity or if PD itself contributed to IAA formation. The use of oral antibiotics post-discharge has not been shown to be associated with IAA formation [35]. Similarly, our study observed that oral antibiotics were administered at comparable rates to patients with IAA and those without, suggesting that oral antibiotics do not prevent IAA.

So, what factors can better predict and early detect the development of IAA? Several studies have proposed the following discharge criteria as useful indicators: (1) afebrile for 24 h, (2) tolerance of a regular diet, (3) manageable abdominal pain, and (4) normalization of WBC count [35,36,37]. Our study found that prolonged postoperative fever occurred significantly more frequently in the IAA group. However, the other three factors were not evaluated in this study. To prevent the occurrence of IAA and improve clinical practice for patients, it will be essential to establish appropriate discharge criteria.

This study is significant in that it applied the AAST-EGS grading system to classify pediatric AA. This system not only provides a more objective diagnosis but also allows for the prediction of clinical outcomes and prognosis. Its broader adoption across more centers is expected in the future. Additionally, this study identified independent risk factors for the occurrence of IAA through multivariate analysis, and these findings should serve as an important reminder to pediatric surgeons when treating patients with these risk factors. Since the median postoperative day for diagnosing IAA was 11 days (range: 5–62), this suggests that pediatric patients who undergo laparoscopic surgery for perforated appendicitis should be followed up differently from those who have undergone surgery for uncomplicated appendicitis. However, we acknowledge certain limitations in our research. Due to its retrospective nature, the study has inherent limitations. Although the three surgeons aligned their preoperative and postoperative management practices, individual differences in surgical techniques likely existed. Furthermore, the relatively small number of enrolled patients is another limitation of this study. Also, this study may have limitations due to potential overlooked cases of appendicolith, which could influence the findings. Given that CT and ultrasound vary in sensitivity and specificity for detecting appendicolith, undetected cases might have impacted the observed incidence rates and associated risk factors in our research.

## 5. Conclusions

Our findings indicate that higher appendicitis severity grades (≥IV) and the intraoperative presence of free appendicolith are significant risk factors for developing IAAs following laparoscopic appendectomy for perforated appendicitis in children. The results highlight the importance of meticulous surgical technique and comprehensive patient assessment in managing pediatric perforated appendicitis. Conducting larger-scale prospective studies could corroborate our findings and facilitate the development of standardized protocols aimed at minimizing postoperative IAA formation.

## Figures and Tables

**Table 1:** Characteristics of the patients (*N* = 137).

Variables	Numbers
Male	76 (55.5%)
Ages (years) *	11.19 (3.75–18.98)
Body weight at operation (kg) *	42 (13–92)
Duration of symptoms to operation (hours) *	41 (10–367)
Severity grading	
III	82 (59.9%)
IV	36 (26.3%)
V	19 (13.8%)
Surgical approach	
Single port	56 (40.9%)
Multiple port	81 (59.1%)
Peritoneal drain	85 (62%)
Operation times (min) *	50 (20–215)
Hospital days after appendectomy *	3 (1–16)
Oral antibiotics at discharge	114 (83.2%)
Complications	28 (20.4%)
Intraabdominal abscess	15 (10.9%)
Complicated fluid collection with mesenteric infiltration	3 (2.2%)
Superficial surgical site infection	10 (7.3%)
Ileus	7 (5.1%)
Bleeding	1 (0.7%)
Readmission	10 (7.3%)
Duration of follow up (days) *	11 (5–375)

* median.

**Table 2:** Univariable analysis of patients with and without intra-abdominal abscess (IAA).

Details	Non-IAA	IAA	*p*-Value
Patient numbers	122 (89.1%)	15 (10.9%)	
A and B hospital	78	10	1.0
C hospital	22	2	
D hospital	22	3	
Male	69 (56.5%)	7 (46.7%)	0.584
Ages (years) *	11.27 (3.75–18.98)	11.16 (6.51–17.78)	0.747
Body weight at operation (kg) *	43 (13–92)	40 (21–76)	0.747
Duration of symptoms to operation (hours) *	38 (10–367)	58 (29–150)	0.012
Preoperative condition			
≥38 °C	78 (63.9%)	12 (80%)	0.262
WBC * (/µL)	15,270 (4000–36,530)	15,690 (7700–23,260)	0.996
CRP * (mg/dL)	4.33 (0.02–29.3)	10.6 (0.33–22.21)	0.019
Appendicolith on imaging study	77 (63.1%)	14 (93.3%)	0.02
Free appendicolith at operation	13 (10.6%)	9 (60%)	<0.001
Severity grading			<0.001
III	80 (65.6%)	2 (13.3%)	
IV	29 (23.8%)	7 (46.7%)	
V	13 (10.6%)	6 (40%)	
Surgical approach, single port	51 (41.8%)	5 (33.3%)	0.523
Peritoneal drain	73 (59.8%)	12 (80%)	0.164
Operation times (min) *	49 (20–215)	70 (35–157)	0.004
Postoperative fever days			
≥37.5 °C, ≥3 days	23 (18.8%)	9 (60%)	0.001
≥38 °C, ≥3 days	9%)	4 (26.7%)	0.037
Hospital days after appendectomy *	3 (1–12)	6 (3–16)	<0.001
Oral antibiotics at discharge	101 (82.8%)	13 (86.7%)	1.0
Complications without IAA	13 (10.6%)	4 (26.7%)	0.093
Readmission	0	9 (60%)	<0.001

* median.

**Table 3:** Multivariable logistic regression analysis of risk factors for intra-abdominal abscess.

Variables	*p*-Value	Odds Ratio	95% CI
Grade of acute appendicitis ≥ IV	0.034	5.9	1.139–30.577
Free appendicolith at operation	0.01	5.549	1.494–20.603

CI; confidence interval.

**Table 4:** Clinical characteristics of patients with intra-abdominal abscess.

Patients	Location	Size	Treatment
1	RLQ	2 × 1.5 cm	IV anti
2	RLQ	2 × 1.2 cm	IV anti
3	Paracolic	3.2 × 1.7 cm	IV anti
4	RLQ, Pelvis	Several, ≤2 cm	IV anti
5	Pelvis	5 × 3.5 cm	IV anti
6	RLQ	3 × 2 cm	IV anti
7	RLQ, LLQ, LUQ, pelvis	Several, ≤1 cm	IV anti
8	RLQ	3.7 × 3.1 cm	IV anti
9	RLQ	4 × 2 cm	IV anti
10	Pelvis	6 × 5 cm	PCD with IV anti
11	RLQ	6 × 5 cm	PCD with IV anti
12	Paracolic	5 × 4 cm	PCD with IV anti
13	Pelvis	5.5 × 4.3 cm	PCD with IV anti
14	RLQ, abscess with remnant appendicolith	3 × 3 cm	Re-operation, POD 62
15	Paracolic, abscess with remnant appendicolith	3 × 2 cm	Re-operation, POD 5

IV anti; intravenous antibiotics, RLQ; right lower quadrant, LLQ; left lower quadrant, LUQ; left upper quadrant, PCD; percutaneous drainage, POD; postoperative day.

## Data Availability

The data presented in this study are available in Supplementary Materials.

## Supplementary Materials

The following supporting information can be downloaded at: https://www.mdpi.com/article/10.3390/children11111385/s1, Table S1: Pediatric laparoscopic appendectomy and intraabdominal abscess.

## References

[1] Di Saverio S., Podda M., De Simone B., Ceresoli M., Augustin G., Gori A., Boermeester M., Sartelli M., Coccolini F., Tarasconi A. (2020). Diagnosis and treatment of acute appendicitis: 2020 update of the WSES Jerusalem guidelines. World J. Emerg. Surg..

[2] Huerta C.T., Courel S.C., Ramsey W.A., Saberi R.A., Gilna G.P., Ribieras A.J., Parreco J.P., Thorson C.M., Sola J.E., Perez E.A. (2023). Nationwide management of perforated pediatric appendicitis: Interval versus same-admission appendectomy. J. Pediatr. Surg..

[3] Omling E., Salö M., Saluja S., Bergbrant S., Olsson L., Persson A., Björk J., Hagander L. (2019). Nationwide study of appendicitis in children. Br. J. Surg..

[4] Mulita F., Plachouri K.-M., Liolis E., Kehagias D., Kehagias I. (2021). Comparison of intra-abdominal abscess formation after laparoscopic and open appendectomy for complicated and uncomplicated appendicitis: A retrospective study. Videosurgery Other Miniinvasive Tech..

[5] Rentea R.M., Peter S.D.S., Snyder C.L. (2017). Pediatric appendicitis: State of the art review. Pediatr. Surg. Int..

[6] Vahdad M.R., Troebs R.-B., Nissen M., Burkhardt L.B., Hardwig S., Cernaianu G. (2013). Laparoscopic appendectomy for perforated appendicitis in children has complication rates comparable with those of open appendectomy. J. Pediatr. Surg..

[7] Mancini G.J., Mancini M.L., Nelson H.S. (2005). Efficacy of laparoscopic appendectomy in appendicitis with peritonitis. Am. Surg..

[8] Jen H.C., Shew S.B. (2010). Laparoscopic versus open appendectomy in children: Outcomes comparison based on a statewide analysis. J. Surg. Res..

[9] Yagmurlu A., Vernon A., Barnhart D.C., Georgeson K.E., Harmon C.M. (2006). Laparoscopic appendectomy for perforated appendicitis: A comparison with open appendectomy. Surg. Endosc..

[10] Vegunta R.K., All A., Wallace L.J., Switzer D.M., Pearl R.H. (2004). Laparoscopic appendectomy in children: Technically feasible and safe in all stages of acute appendicitis. Am. Surg..

[11] Van Amstel P., The S.M.L., Mulder I.M., Bakx R., Derikx J.P.M., van Schuppen J., de Vries R., van der Kuip M., Zijp G.W., Allema J.H. (2022). The Management of Post-appendectomy Abscess in Children; A Historical Cohort Study and Update of the Literature. Front. Pediatr..

[12] Van den Boom A.L., Gorter R.R., van Haard P.M.M., Doornebosch P.G., Heij H.A., Dawson I. (2015). The impact of disease severity, age and surgical approach on the outcome of acute appendicitis in children. Pediatr. Surg. Int..

[13] St Peter S.D., Sharp S.W., Holcomb G.W., Ostlie D.J. (2008). An evidence-based definition for perforated appendicitis derived from a prospective randomized trial. J. Pediatr. Surg..

[14] Emil S., Elkady S., Shbat L., Youssef F., Baird R., Laberge J.-M., Puligandla P., Shaw K. (2014). Determinants of postoperative abscess occurrence and percutaneous drainage in children with perforated appendicitis. Pediatr. Surg. Int..

[15] Tominaga G.T., Staudenmayer K.L., Shafi S., Schuster K.M., Savage S.A., Ross S., Muskat P., Mowery N.T., Miller P., Inaba K. (2016). The American Association for the Surgery of Trauma grading scale for 16 emergency general surgery conditions: Disease-specific criteria characterizing anatomic severity grading. J. Trauma Acute Care Surg..

[16] Gasior A.C., Marty Knott E., Ostlie D.J., St Peter S.D. (2013). To drain or not to drain: An analysis of abscess drains in the treatment of appendicitis with abscess. Pediatr. Surg. Int..

[17] Ferris M., Quan S., Kaplan B.S., Molodecky N., Ball C.G., Chernoff G.W., Bhala N., Ghosh S., Dixon E., Ng S. (2017). The Global Incidence of Appendicitis: A Systematic Review of Population-based Studies. Ann. Surg..

[18] Oh C., Lee S., Chang H.K., Ahn S.M., Chae K., Kim S., Kim S., Seo J.-M. (2021). Analysis of Pediatric Surgery Using the National Healthcare Insurance Service Database in Korea: How Many Pediatric Surgeons Do We Need in Korea?. J. Korean Med. Sci..

[19] Newman K., Ponsky T., Kittle K., Dyk L., Throop C., Gieseker K., Sills M., Gilbert J. (2003). Appendicitis 2000: Variability in practice, outcomes, and resource utilization at thirty pediatric hospitals. J. Pediatr. Surg..

[20] Collins C.M., Davenport D.L., Talley C.L., Bernard A.C. (2018). Appendicitis Grade, Operative Duration, and Hospital Cost. J. Am. Coll. Surg..

[21] Markar S.R., Blackburn S., Cobb R., Karthikesalingam A., Evans J., Kinross J., Faiz O. (2012). Laparoscopic versus open appendectomy for complicated and uncomplicated appendicitis in children. J. Gastrointest. Surg..

[22] Aziz O., Athanasiou T., Tekkis P.P., Purkayastha S., Haddow J., Malinovski V., Paraskeva P., Darzi A. (2006). Laparoscopic versus open appendectomy in children: A meta-analysis. Ann. Surg..

[23] Inagaki K., Blackshear C., Morris M.W., Hobbs C.V. (2020). Pediatric Appendicitis-Factors Associated With Surgical Approach, Complications, and Readmission. J. Surg. Res..

[24] Nataraja R.M., Teague W.J., Galea J., Moore L., Haddad M.J., Tsang T., Khurana S., Clarke S.A. (2012). Comparison of intraabdominal abscess formation after laparoscopic and open appendicectomies in children. J. Pediatr. Surg..

[25] Kaewlai R., Wongveerasin P., Lekanamongkol W., Wongsaengchan D., Teerasamit W., Tongsai S., Khamman P., Chatkaewpaisal A., Noppakunsomboon N., Apisarnthanarak P. (2024). CT of appendicoliths in adult appendicitis: Clinical significance and characteristics of overlooked cases. Eur. Radiol..

[26] Monsonis B., Mandoul C., Millet I., Taourel P. (2020). Imaging of appendicitis: Tips and tricks. Eur. J. Radiol..

[27] Blumfield E., Nayak G., Srinivasan R., Muranaka M.T., Blitman N.M., Blumfield A., Levin T.L. (2013). Ultrasound for differentiation between perforated and nonperforated appendicitis in pediatric patients. AJR Am. J. Roentgenol..

[28] Gonzalez D.O., Lawrence A.E., Cooper J.N., Sola R., Garvey E., Weber B.C., St Peter S.D., Ostlie D.J., Kohler J.E., Leys C.M. (2018). Can ultrasound reliably identify complicated appendicitis in children?. J. Surg. Res..

[29] Lowe L.H., Penney M.W., Scheker L.E., Perez R., Stein S.M., Heller R.M., Shyr Y., Hernanz-Schulman M. (2000). Appendicolith revealed on CT in children with suspected appendicitis: How specific is it in the diagnosis of appendicitis?. AJR Am. J. Roentgenol..

[30] Singh A.K., Hahn P.F., Gervais D., Vijayraghavan G., Mueller P.R. (2008). Dropped appendicolith: CT findings and implications for management. AJR Am. J. Roentgenol..

[31] Betancourt S.L., Palacio D., Bisset G.S. (2015). The ‘wandering appendicolith’. Pediatr. Radiol..

[32] Bi L.-W., Yan B.-L., Yang Q.-Y., Cui H.-L. (2019). Peritoneal irrigation vs suction alone during pediatric appendectomy for perforated appendicitis: A meta-analysis. Medicine.

[33] Escolino M., Becmeur F., Saxena A., Till H., Masieri L., Cortese G., Holcomb G.W., Esposito C. (2018). Infectious Complications After Laparoscopic Appendectomy in Pediatric Patients with Perforated Appendicitis: Is There a Difference in the Outcome Using Irrigation and Suction Versus Suction Only? Results of a Multicentric International Retrospective Study. J. Laparoendosc. Adv. Surg. Tech..

[34] Narci A., Karaman I., Karaman A., Erdoğan D., Cavuşoğlu Y.H., Aslan M.K., Cakmak O. (2007). Is peritoneal drainage necessary in childhood perforated appendicitis?--a comparative study. J. Pediatr. Surg..

[35] Gordon A.J., Choi J.-H., Ginsburg H., Kuenzler K., Fisher J., Tomita S. (2020). Oral Antibiotics and Abscess Formation After Appendectomy for Perforated Appendicitis in Children. J. Surg. Res..

[36] Svetanoff W.J., Talukdar N., Dekonenko C., Dorman R.M., Osuchukwu O., Fraser J.D., Oyetunji T.A., St Peter S.D. (2020). Intra-abdominal Abscess After Appendectomy-Are Drains Necessary in All Patients?. J. Surg. Res..

[37] Nielsen J.W., Kurtovic K.J., Kenney B.D., Diefenbach K.A. (2016). Postoperative timing of computed tomography scans for abscess in pediatric appendicitis. J. Surg. Res..

